# ALZHEIMER’S DISEASE AND RELATED DEMENTIAS IN A FAMILY CONTEXT

**DOI:** 10.1093/geroni/igad104.0053

**Published:** 2023-12-21

**Authors:** J Jill Suitor, Megan Gilligan

## Abstract

This symposium considers the ways in which the presence of Alzheimer’s Disease and Related Dementias (ADRD) shapes functional and affective relationships between family members, and raises questions about the future public health implications of ADRD family caregiving. In the first paper, Huo and Kim use dyadic data collected from husbands and wives in which one partner lives with mild-to-moderate cognitive AD to explore the role of relationship quality in caregiver stress. Next, Suitor and colleagues use mixed-methods data from the Within-Family Differences Study to examine how mothers’ cognitive impairment moderates the impact of maternal favoritism/disfavoritism on the psychological well-being of adult sons and daughters. Third, Gilligan, Suitor and Ogle investigate the role of adult children’s care provision in the association between parents’ cognitive impairment and adult sibling tension, also using mixed-methods data from the WFDS. Next, Savla and Roberto use mixed-methods data to explore how extended family caregivers manage their care responsibilities and how care arrangements impact the provision of future care for persons living with ADRD. Finally, Gaugler considers the public health ramifications of family caregiving, summarizing the scientific literature on estimating the future supply of family caregivers, and presenting a research agenda to address the potential family care gap. Taken together, this diverse set of papers highlights the complex ways in which the presence of ADRD shapes patterns of care, relationship quality, and well-being of members of later-life families. This is a collaborative symposium between the Alzheimer’s Disease and Related Dementias and Family Caregiving Interest Groups.

